# β-Cyclodextrin Derivative Grafted on Silica Gel Represents a New Polymeric Sorbent for Extracting Nitisinone from Model Physiological Fluids

**DOI:** 10.3390/molecules26195945

**Published:** 2021-09-30

**Authors:** Magdalena Danek, Anna Korytkowska-Wałach, Hanna Barchańska

**Affiliations:** 1 Department of Inorganic Chemistry, Analytical Chemistry and Electrochemistry, Faculty of Chemistry, Silesian University of Technology, B. Krzywoustego 6 St., 44-100 Gliwice, Poland; Hanna.Barchanska@polsl.pl; 2 Department of Organic Chemistry, Bioorganic Chemistry and Biotechnology, Faculty of Chemistry, Silesian University of Technology, B. Krzywoustego 4 St., 44-100 Gliwice, Poland; Anna.Korytkowska-Walach@polsl.pl

**Keywords:** nitisinone, β-cyclodextrin, nuclear magnetic resonance, Fourier transform infrared spectroscopy, model physiological fluids, solid phase extraction, liquid chromatography

## Abstract

Nitisinone (NTBC) is used in the treatment of disorders affecting the tyrosine pathway, including hereditary tyrosinemia type I, alkaptonuria, and neuroblastoma. An inappropriate dosage of this therapeutic drug causes side effects; therefore, it is necessary to develop a rapid and sensitive method to monitor the content of NTBC in patients’ blood. This study aimed to develop anew polymeric sorbent containing β-cyclodextrin (β-CD) derivatives grafted on silica gel to effectively extract NTBC from model physiological fluids. The inclusion complex formed between β-CD and NTBC was examined by proton nuclear magnetic resonance spectroscopy. The novel sorbents with derivatives of β-CD were prepared on modified silica gel using styrene as a comonomer, ethylene glycol dimethacrylate as a crosslinking agent, and 2,2′-azo-bis-isobutyronitrile as a polymerization initiator. The obtained products were characterized via Fourier transform infrared spectroscopy and then used as sorbents as part of a solid phase extraction technique. High NTBC recovery (70%indicated that the developed polymeric sorbent may be suitable for extracting this compound from patients’ blood samples.

## 1. Introduction

Nitisinone (2-(2-nitro-4-trifluoromethylbenzoyl)cyclohexane-1,3-dione; NTBC) represents a functional triketone compound. Toxicology tests of NTBC involving rats have shown that it is an inhibitor of the enzyme, hydroxyphenylpyruvate dioxygenase (HPPD), which participates in tyrosine metabolism. This discovery inspired further research into the potential implementation of this compound as a drug for treating various disorders affecting the tyrosine pathway [1,2,3].

NTBC was approved for use as a drug by the United States Food and Drug Administration and the European Union Drug Agency in 2002 and 2005, respectively [4,5]. This drug is marketed as Orfadin^®^ and is used in the treatment of hereditary tyrosinemia type I (HT1), which is a rare inborn metabolic disorder that is characterized by a deficiency of fumarylacetoacetate during the metabolism of tyrosine; this leads to an increase in the concentration of this amino acid and other compounds that can be toxic for humans. The application of NTBC reduces the production of toxic metabolites by effectively blocking the tyrosine metabolic pathway [4,5,6,7]. NTBC can be also used in the treatment of alkaptonuria [8,9] or neuroblastoma [4,10].

Patients undergoing an NTBC treatment process may experience side effects; therefore, it is crucial to select the safest and most appropriate therapeutic dose. To do so, it is necessary to employ a quick and sensitive method, which involves continuously monitoring the patient’s metabolism, controlling the concentration of NTBC in physiological fluids, and appropriately adjusting the drug dose [8,11,12].

The elaboration of an analytical method requires several key steps, including sampling, pretreatment, transport, storage, sample preparation, final analysis, and analytical data processing. However, the most important step in this approach is the sample preparation because it has a significant impact on the results of the final analysis. This step may involve various extraction techniques, which enable: (i) the extraction of the analyte(s); (ii) preconcentration; and (iii) purification of the sample [13,14,15,16,17].

Statistics indicate that the most commonly used extraction technique in analytical chemistry is solid phase extraction (SPE) [18]. The SPE procedure involves conditioning the sorbent, loading the sample, removing any interfering (matrix) compounds, and eluting the analyte [13]. Selecting an appropriate sorbent according to the physicochemical properties of the analyte and matrix compounds allows for high recovery of the desired analyte and effective clean-up of the sample. Sorbents used in SPE generally belong to three groups: inorganic oxides (e.g., silica gel), low-specificity sorbents (e.g., octadecyl sorbent, C18; polymeric sorbent styrene-divinylbenzene, HLB), and specific sorbents (e.g., molecularly imprinted polymers, MIPs) [19,20,21].

Recently, there has been increased interest regarding the application of polymeric sorbents based on cyclodextrin (CD) [22,23,24,25]. CDs are cyclic oligosaccharides that can be obtained via the enzymatic degradation of starch. Moreover, they are low-cost natural materials that do not pose a risk to the environment [25,26]. In general, CDs are composed of six or more D-glucopyranoside units linked via α(1–4); native CDs contain six (α-CD), seven (β-CD), or eight (γ-CD) units of glucose. The most frequently used CD is β-CD owing to its cost-effectiveness, wide availability, and capacity to form inclusion compounds with various substances through host–guest interactions. The central portion of β-CD contains seven units of glucose and exhibits hydrophobic properties, whereas the external part includes hydroxyl groups and is hydrophilic. This unique structure allows facile encapsulation of various compounds, and modifications of the β-CD surface can expand its ability to encapsulate compounds characterized by diverse physicochemical properties [26]. Moreover, the bonding of the β-CD to the support material (e.g., silica gel) increases the adsorption properties of the polymeric sorbents based on CD [27,28,29,30].

To develop new sorbents, it is necessary to estimate the interactions between the host (e.g., functional monomer) and guest (e.g., analyte); such evaluations are typically carried out using proton nuclear magnetic resonance spectroscopy (^1^H-NMR), which can provide key information about the host–guest interaction. This capability originates from the fact that the formation of a host–guest complex induces changes in the electron densities within the functional monomer and the analyte, which leads to observable changes in the proton chemical shifts [31,32].

A comprehensive literature review revealed that dried blood spot (DBS) testing is the sole technique used to determine the NTBC concentration in patient blood samples [12,33,34,35,36]. Therefore, it is necessary to find new analytical methods to provide an alternative to DBS.

The present study aimed to develop a new polymeric sorbent containing a derivative of β-CD grafted on silica gel for the extraction of NTBC from model physiological fluids. The interactions between β-CD and NTBC were evaluated using ^1^H-NMR, and the obtained sorbent was characterized by Fourier transform infrared spectroscopy (FTIR). The applied analytical method based on SPE with the new polymeric sorbent was validated. Overall, the results presented herein indicate that the developed sorbent can be used to effectively extract NTBC from patient blood samples.

## 2. Results and Discussion

### 2.1. Characterization of β-CD/NTBC Complexes by NMR

An aqueous solution of 3-(trimethylsilyl)propionic-2,2,3,3-d_4_ acid sodium salt (TSP) was used as an external reference to avoid errors in the determination of association constants [37].

#### 2.1.1. Continuous Variation Method for Determining Stoichiometry

First, the NMR characterization of the NTBC/β-CD complexation involved determining the stoichiometry of the formed complex.

**Figure 1:**
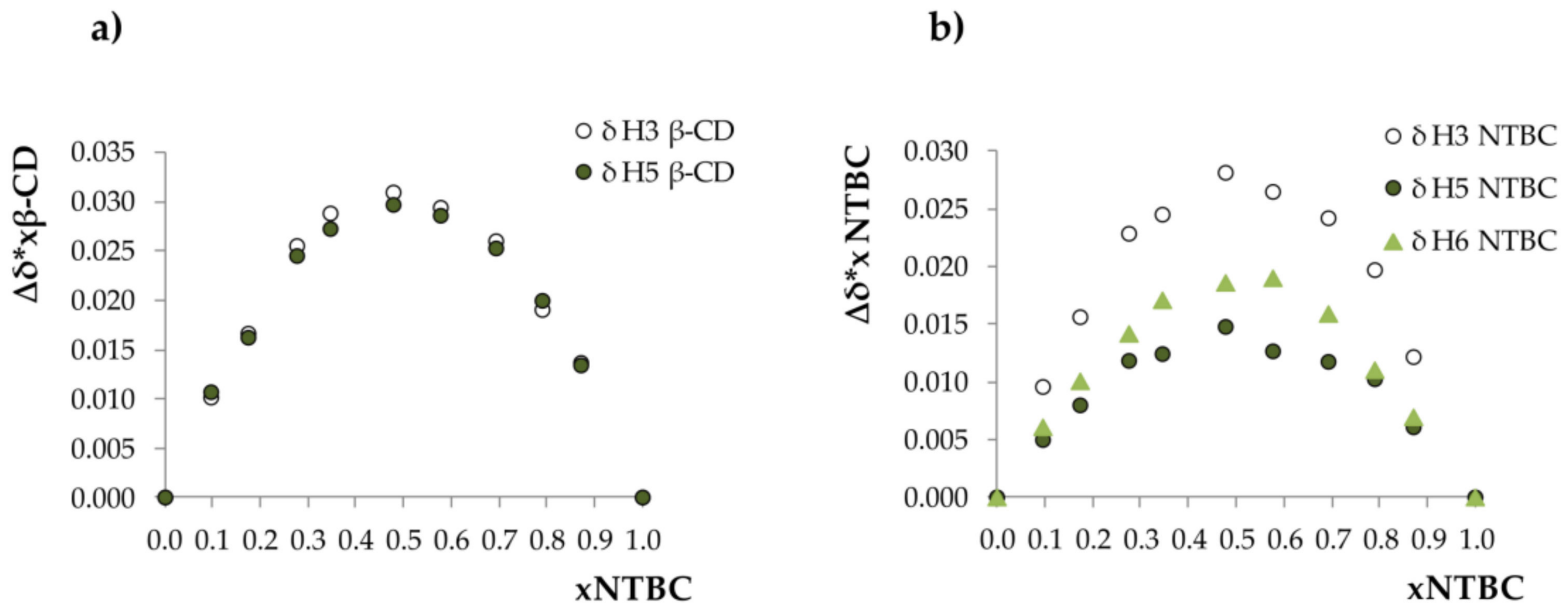
Job’s plots for β-CD/NTBC constructed according to the chemical shifts of (**a**) protons H^3^ and H^5^ of β-CD, and (**b**) the aromatic protons of NTBC.

Using a continuous variation method, the stoichiometry of the generated complex was determined. The Job’s plots (Figure 1) constructed based on the chemical shifts of corresponding protons are both characterized by a sharp symmetrical shape, with a maximum at mole fraction of nitisinone xNTBC = 0.5, which corresponds to 1:1 stoichiometry of the complex formed.

#### 2.1.2. 2D-ROESY

**Figure 2:**
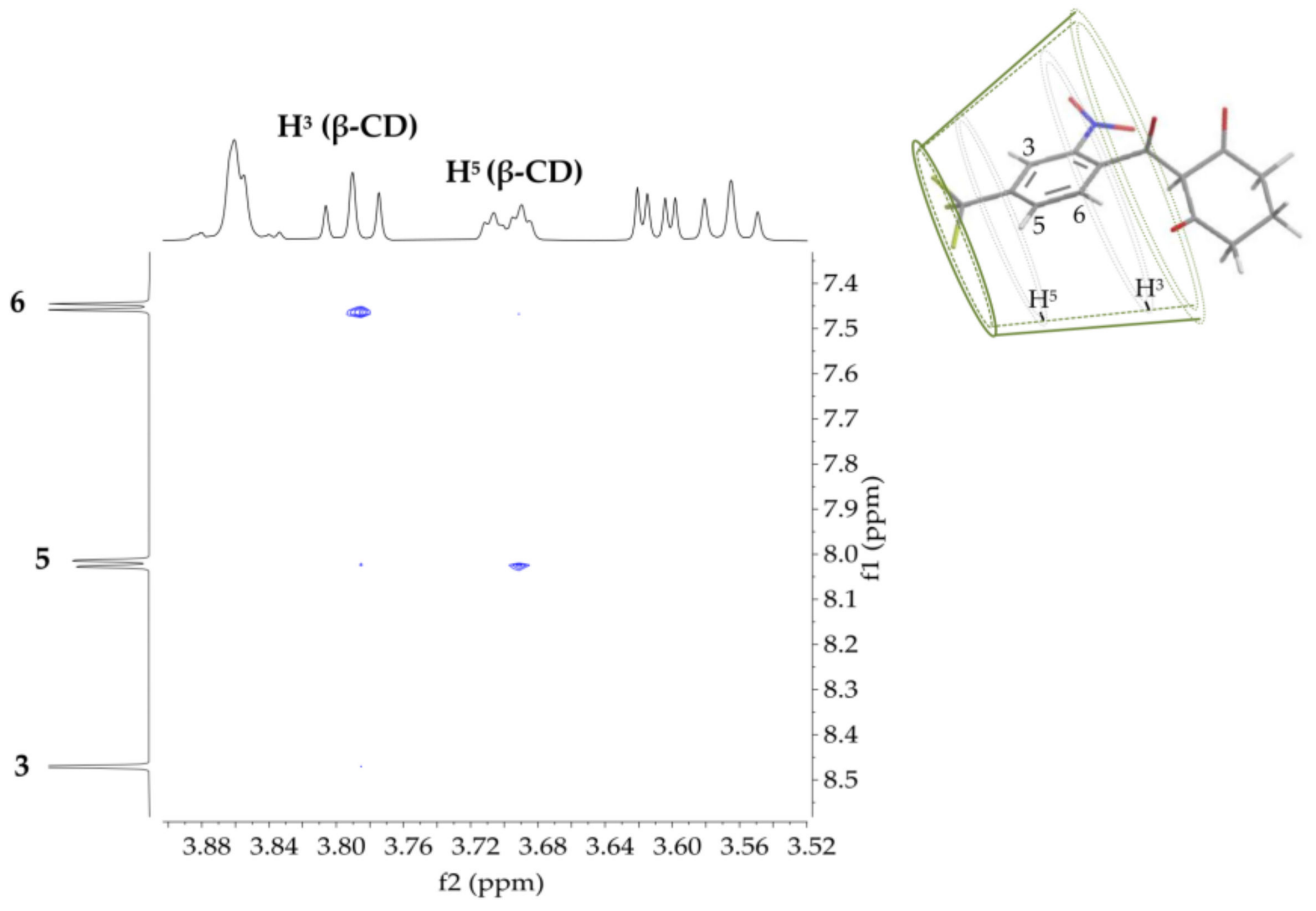
Proposed geometry of the β-CD/NTBC inclusion complex and a partial contour plot of the 2D-ROESY spectrum recorded for a 4:1 β-CD/NTBC ratio.

Two-dimensional rotating-frame Overhauser effect spectroscopy (2D ROESY) was used to confirm the formation of the inclusion complex. A four-fold excess of NTBC was introduced to shift the complexation reaction toward complex formation. Figure 2 shows the correlation signals, which indicate the close proximity between H^3^ of β-CD and H^6^ of NTBC and between H^5^ of β-CD and H^5^ of NTBC. The proposed orientation of the NTBC molecule in the cyclodextrin cavity is also presented.

#### 2.1.3. Titrations to Obtain Association Constants

**Figure 3:**
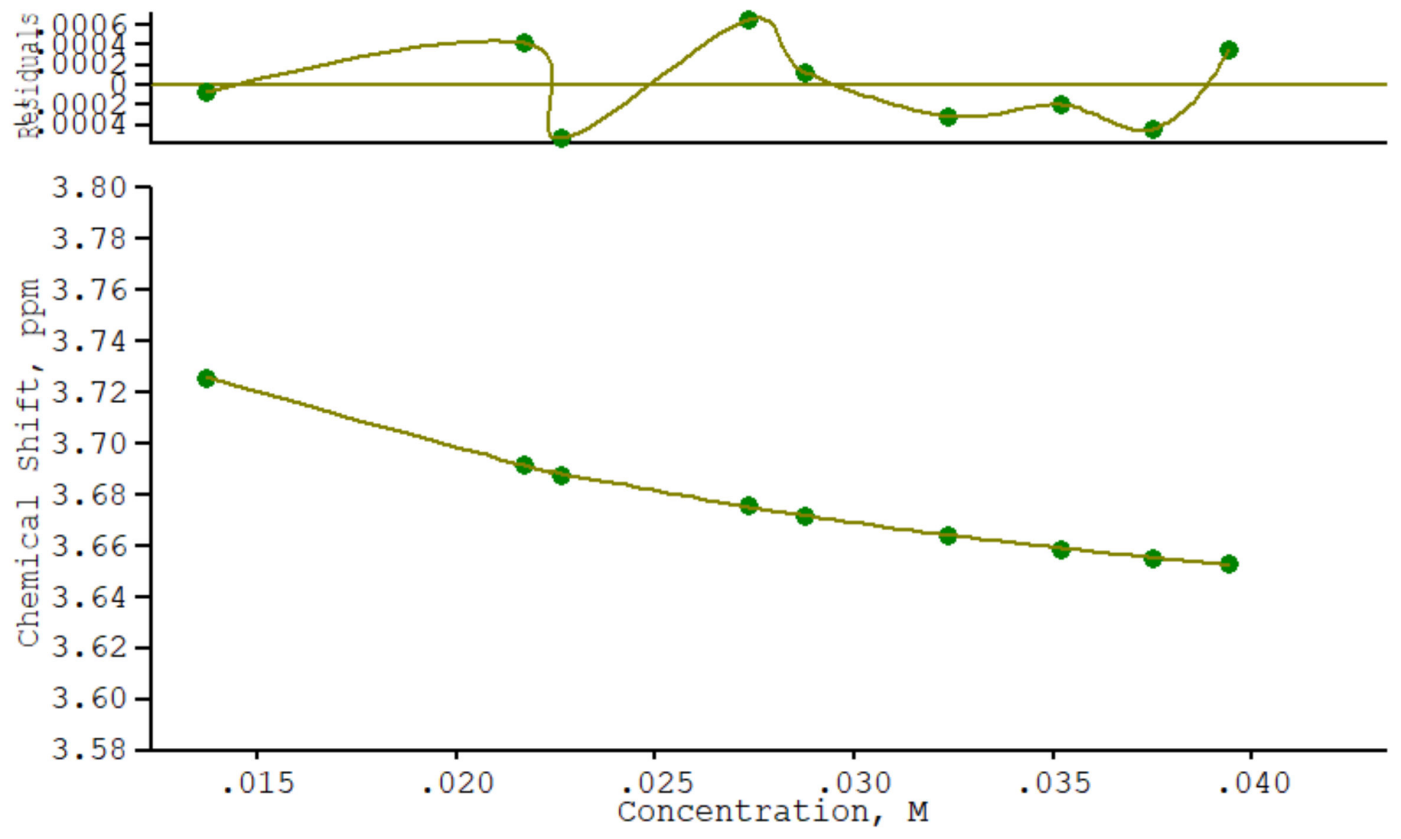
The chemical shift of the H^3^ proton of β-CD as a function of the NTBC concentration and the experimental data fitting (by WinEQNMR) for the NMR titration of β-CD with NTBC (a magnification of the residuals is also presented).

**Figure 4:**
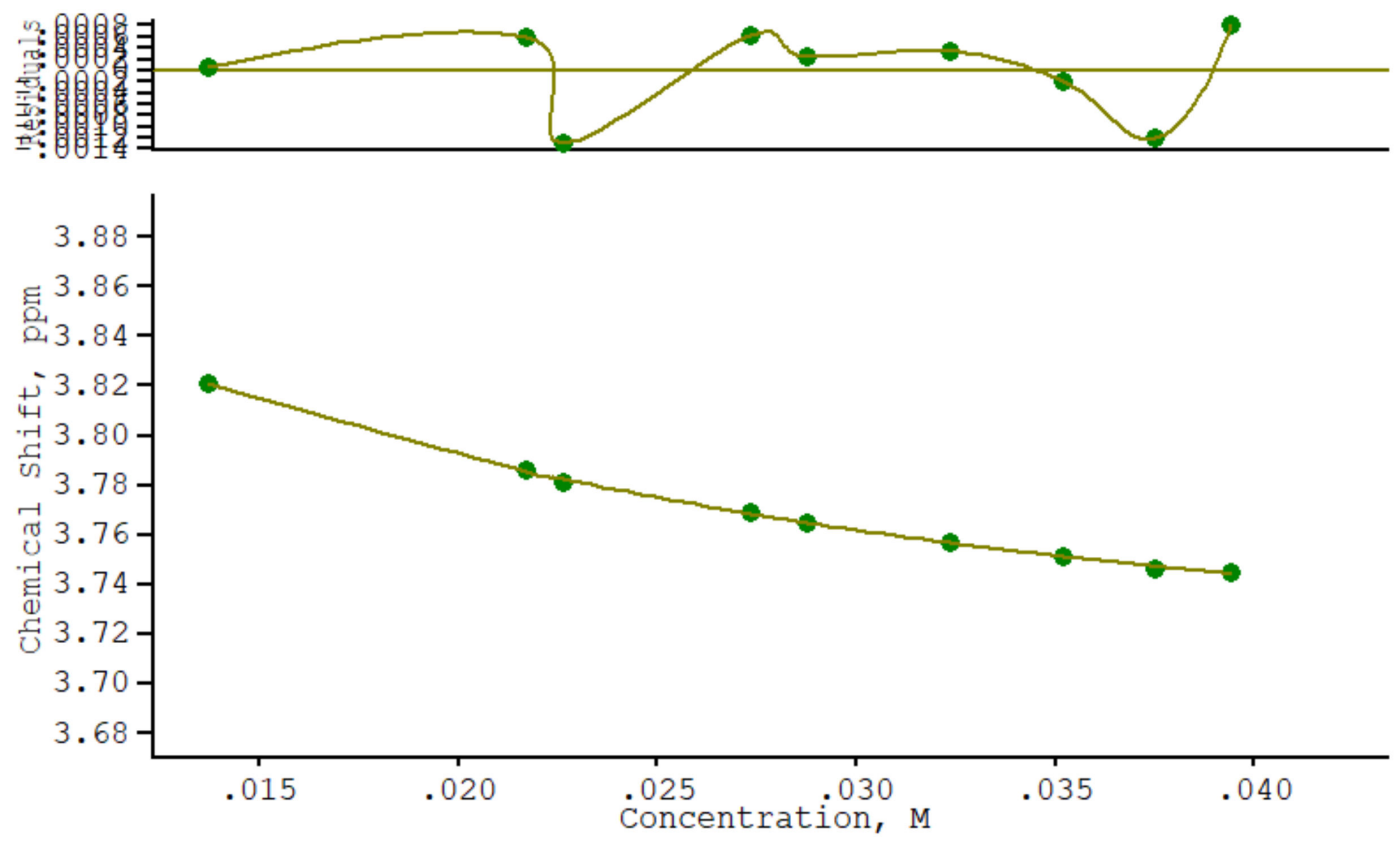
The chemical shift of the H^5^ proton of β-CD as a function of the NTBC concentration and the experimental data fitting (by WinEQNMR) for the NMR titration of β-CD with NTBC (a magnification of the residuals is also presented).

The association constant governing NTBC/β-CD complex formation was determined from NMR titration experiments (i.e., measuring changes in chemical shifts as a function of the concentration of one reagent). For these experiments, the β-CD concentration was held constant, and the concentration of NTBC was increased incrementally to cover host:guest ratios from 0.0 to 4.0. An upfield shift of the signals corresponding to the inner protons of β-CD (i.e., H^3^ and H^5^) was observed as the concentration of NTBC increased. The collected NMR data were fitted using a curve-fitting method within the WinEQNMR computer program (Figure 3 and Figure 4). This program applied non-linear least-squares subroutines that carried out refinements of various parameters according to the Levenberg–Marquardt method, which does not require that one reactant exists in a large excess [38].

The calculated association constants (within the error given by WinEQNMR2) based on the chemical shifts of the H^3^ and H^5^ signals of β-CD are 85.5 (8.5) M^−1^ and 94.4 (6.0) M^−1^, respectively. Individual association constant values differ by about 10%. Good agreement between the association constants K determined from the two β-CD signals, as well as the absence of systematic deviations in this case, confirms the assumed 1:1 intracavity complexation and that no other supramolecular species are present. The equilibrium individual association constants obtained from NMR titration corresponds to a Gibbs free energy ΔG = −11 kJ/mol. ΔG values are negative, which indicates that inclusion process proceed spontaneously at 25 °C.

The results of these NMR studies of the complexation of NTBC by β-CD clearly confirm that inclusion complexes are formed. Therefore, it was concluded that the incorporation of β-CD molecules on the surface of the sorbent should improve its adsorption properties towards NTBC molecules.

### 2.2. Preparation and Characterization of Sorbents

A radical polymerization method was used to attach functional monomers bearing β-CD moieties onto silica gel in order to prepare a sorbent containing surface β-CDs for the complexation of NTBC. In the first step of this process, the silica surface was prepared by introducing bonds conducive to free radical polymerization.

In this case, 3-(Trimethoxysilyl)propyl methacrylate (γ-MAPS) containing -Si(-OCH_3_)_3_ groups, capable of reacting with hydroxyl groups of the silica surface, was used. Thus, methacrylate bonds of γ-MAPS are able for free radical polymerization with functional β-CD monomers.

**Scheme 1:**
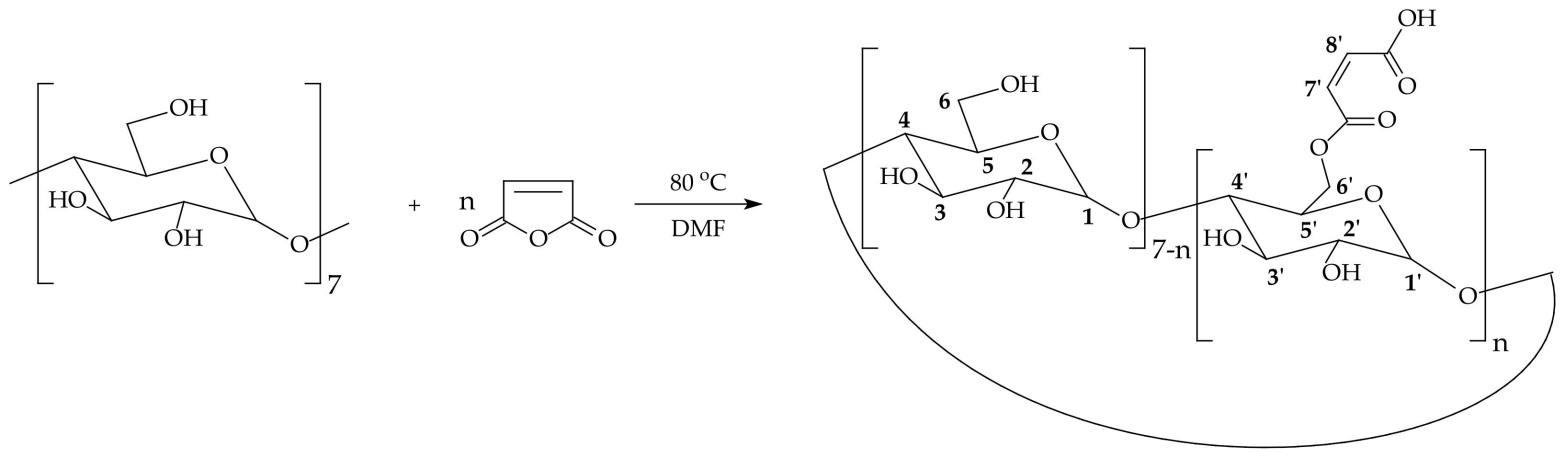
Addition of maleic anhydride to β-cyclodextrin.

The functional monomers bearing β-CD moieties were synthesized according to a modified version of the procedure reported by Liu and Fan [39], which involved the simple addition of maleic anhydride to β-CD (Scheme 1). By using an appropriate molar ratio of maleic anhydride to cyclodextrin and an appropriate reaction time, a functional monomer was obtained. According to ^1^H-NMR analysis, each monomer contains an average of 1.14 molecules of maleic anhydride per one molecule of CD. It was presumed that this number of maleic moieties would not reduce the tendency of CD to form inclusion complexes, but rather, it ensured the incorporation of the functional monomer into the polymer network.

**Scheme 2:**
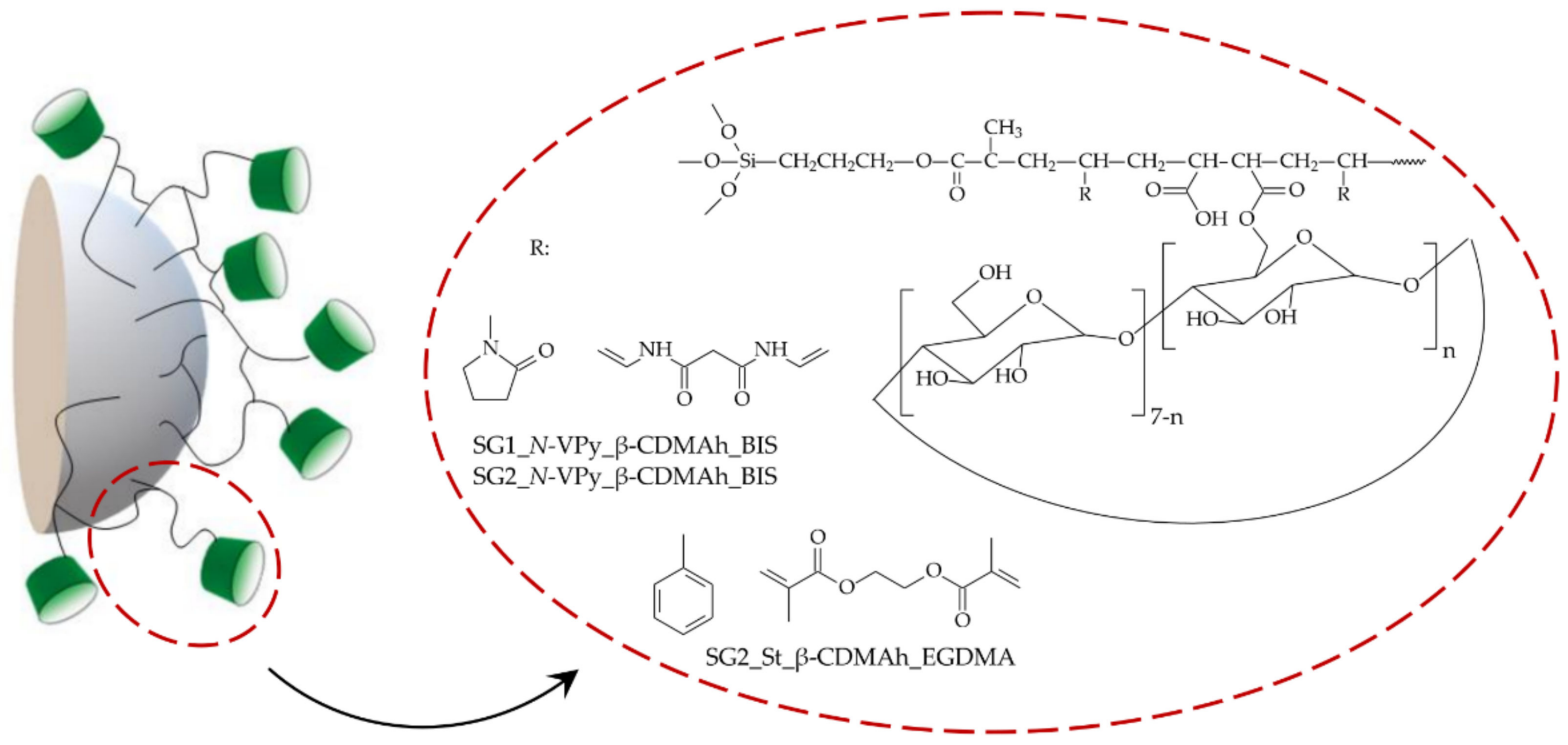
The structure of the sorbents used in this work.

It is well-known that maleic anhydride and its derivatives do not undergo free radical homopolymerization owing their electron-accepting nature. Therefore, it is necessary to use a second comonomer with electron-donating character. In this work, two types of supports were synthesized, wherein either hydrophilic *N*-vinylpyrrolidone (*N*-VPy) or hydrophobic styrene was used as the comonomer. Recently, it has been shown that the free radical polymerization of *N*-VPy with a maleic derivative allows the preparation of polymers containing up to 70 wt.% of CD units [40]. Styrene, on the other hand, is known to form an alternating copolymer when used as a comonomer with maleic anhydride. A crosslinking agent (i.e., *N*,*N*′-methylenebisacrylamide (BIS) or ethylene dimethacrylate (EGDMA)) was used to reduce the mobility of the polymer chains. Scheme 2 shows the structure of the sorbents used in this work.

**Figure 5:**
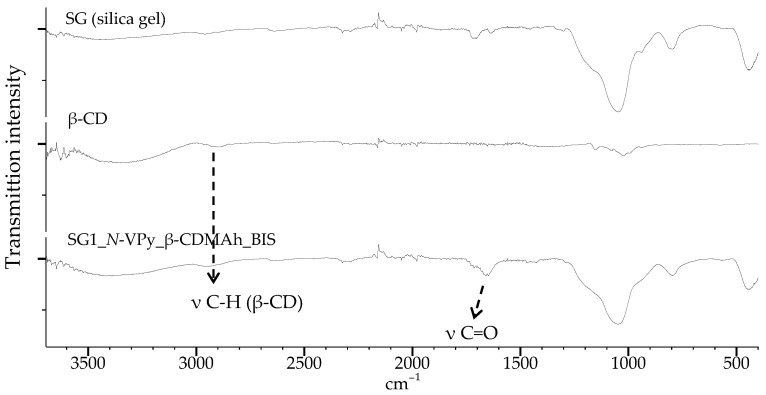
ATR-IR spectra: (top) silica gel; (middle) β-CD; (bottom) SG1_N-VPy_β-CDMAh_BIS.

The obtained sorbents were characterized by attenuated total reflectance (ATR) IR spectroscopy. Figure 5 shows the spectrum of a sorbent prepared by copolymerizing a functional monomer with *N*-VPy on activated silica gel in the presence of BIS as the crosslinking agent using method 1 (SG1_*N*-VPy_β-CDMAh_BIS), which was detailed described in Section 3.4.

**Figure 6:**
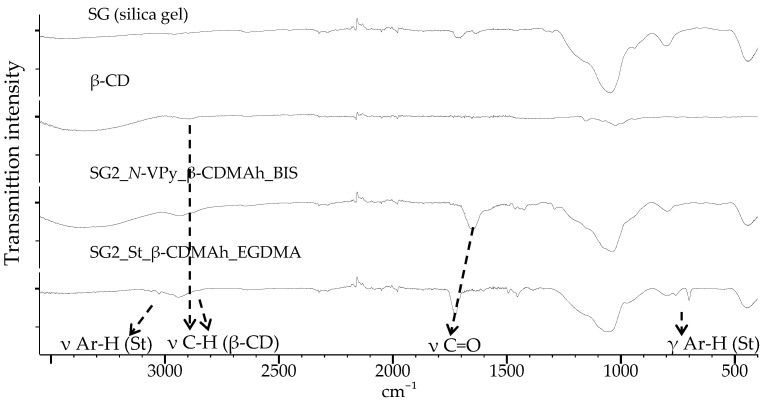
ATR-IR spectra (from the top-down): silica gel; β-CD; SG2_*N*-VPy_β-CDMAh_BIS; SG2_St_β-CDMAh_EGDMA.

The IR spectrum of an analogous polymer on silica gel modified using method 2 (SG2_*N*-VPy_β-CDMAh_BIS) is shown in Figure 6.

Comparing these two spectra reveals that the ratio of the intensities of the polymer peaks to the silica gel signals is higher for the sorbent where the polymer was adhered on the silica gel activated via method 2. It can therefore be assumed that the second method of silica gel activation is more effective and allows a greater amount of the polymer to be introduced on the silica surface. Therefore, this approach was applied to prepare the second type of sorbent, which contained styrene units in addition to cyclodextrin units (SG2_St_β-CDMAh_EGDMA).

In all IR spectra of the obtained sorbents (Figure 5 and Figure 6), a weak band originating from the C–H stretching vibrations in the β-CD molecules appears at about 2883 cm^−1^ (for the SG2_St_β-CDMAh_EGDMA sorbent this band is shifted slightly toward smaller wave numbers). In addition, characteristic signals from the stretching vibrations of carbonyl bonds are visible. For SG2_St_β-CDMAh_EGDMA sorbent, characteristic signals indicating the presence of an aromatic ring are visible.

### 2.3. Recovery Study

The obtained sorbents were used in the SPE technique for the extraction of NTBC from a buffer solution. The first step in SPE was conditioning of the sorbent packed in the SPE cartridge, which involves washing the sorbent with a solvent to prepare it for efficient extraction of NTBC from solution. In the next step, the NTBC solution prepared in buffer ($c_{\mathrm{NTBC}}=20\;\mu g/\mathrm{mL}$, pH = 8.00) was loaded to the SPE cartridge with the sorbent. The concentration of NTBC corresponded to the content of this drug in patients’ blood samples, and the pH of the prepared solution corresponded to the pH of the blood samples. Then, analyte was eluted with the appropriate solvent. During the SPE process, effluents (after the sample loading step) and eluates (after the analyte elution step) were collected to estimate the adsorption of NTBC onto the prepared sorbents.

**Figure 7:**
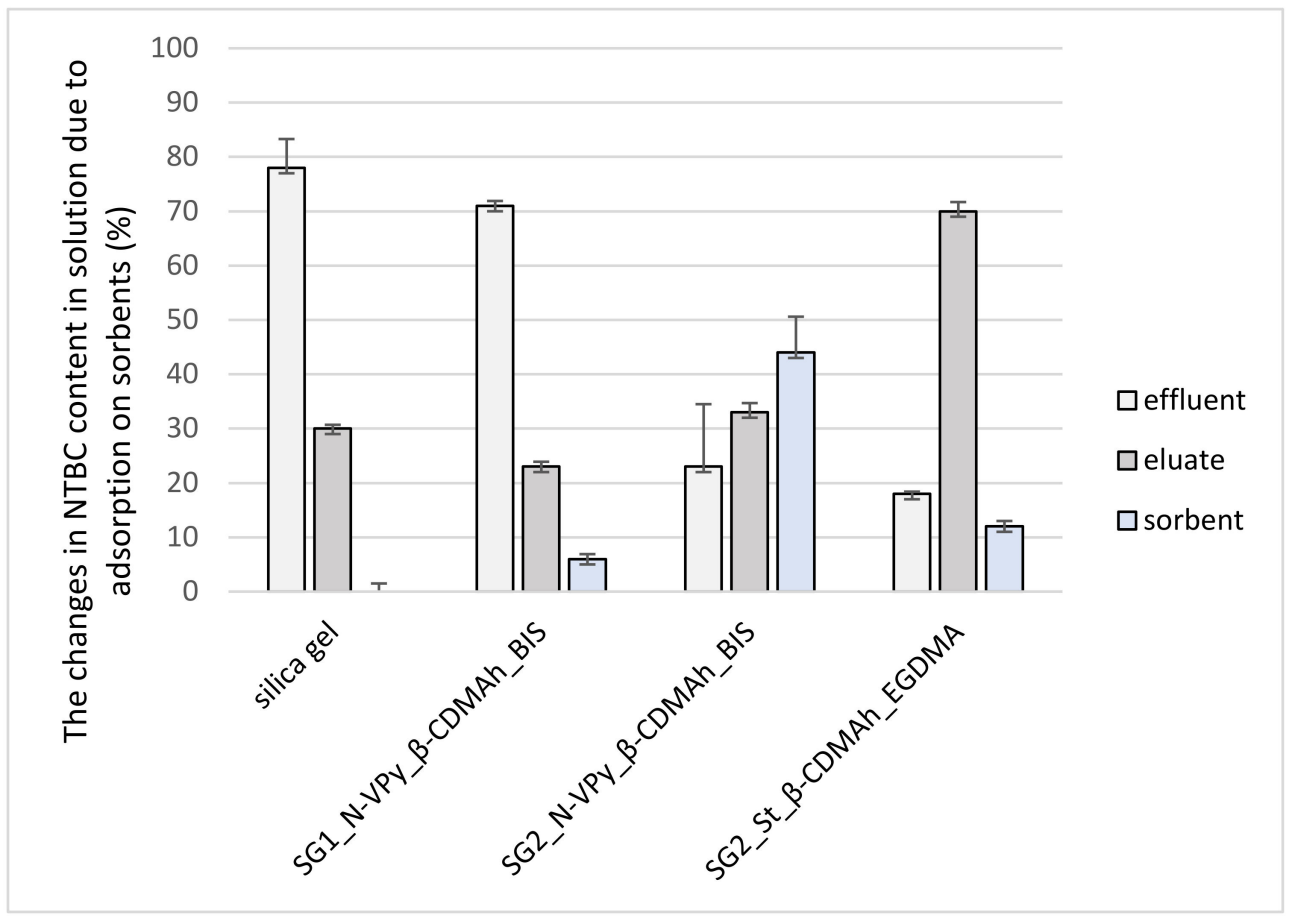
Changes in NTBC content in solution following adsorption to commercially available silica gel and the developed sorbents.

The recoveries (the content of NTBC in the eluate) and the changes in NTBC content in the effluent obtained for commercially available silica gel and the prepared sorbents are presented in Figure 7 with their relative standard deviations (RSD).

The NTBC recovery achieved by silica gel was low at 30%. Simultaneously, the high content of NTBC was determined in the effluent, which indicated weak interactions between the NTBC compound and this sorbents. The low recovery for polar silica gel may be explained by the fact that NTBC, as non-polar compound (water solubility = 0.008 g/L; logP = 1.6), poorly interacts with sorbent characterized by polar properties.

The silica gel was activated with two methods. For the first one, SG1_N-VPy_β-CDMAh_BIS sorbent was obtained, while for the second-SG2_N-VPy_β-CDMAh_BIS. Importantly, the NTBC recovery by SG2_N-VPy_β-CDMAh_BIS was higher than that of SG1_N-VPy_β-CDMAh_BIS (33 and 23%, respectively). In the case of the SG1_N-VPy_β-CDMAh_BIS sorbent, the low recovery of NTBC may result from low effectiveness of the silica gel activation, which did not allow for the introduction of polymer on the silica surface (Section 2.2). Therefore, the high content of NTBC was determined in the effluent for this sorbent.

Moreover, high adsorption of NTBC on the sorbent’s surface (44%) was observed for SG2_N-VPy_β-CDMAh_BIS. This observation may be due to the low elution strength of the solvent. Considering the low introduction of polymer on the silica surface for SG1_N-VPy_β-CDMAh_BIS in comparison to SG2_N-VPy_β-CDMAh_BIS, the second method of modifying the silica gel surface was used for further studies (Section 2.2).

The highest analyte recovery (70%) was observed for SG2_St_β-CDMAh_EGDMA (activation of silica gel with the second method), which indicated strong interactions between NTBC and this sorbent. The introduction of styrene increased the hydrophobic properties of the β-CD surface. The resulting strong hydrophobic interactions between the sorbent’s surface and the analyte, which is a non-polar compound, lead to strong adsorption of the analyte on the sorbent.

The experimental results demonstrated high precision (i.e., RSD in the range: 0.3–11.5%). Based on the analysis of variance (ANOVA) performed using the MATLAB software, the obtained results show statistically significant differences between particular sorbents (*p* < 0.05).

### 2.4. Validation Study

The developed method involving the novel sorbent SG2_St_β-CDMAh_EGDMA were validated in terms of linearity, sensitivity (i.e., limit of quantification, LOQ), accuracy (i.e., recovery, RE), precision (i.e., RSD), and matrix effects (ME). The detailed validation parameters of the elaborated method are presented in Table 1.

A calibration curve prepared with solutions covering the concentration range 1.0–25.0 μg mL^−1^ revealed that NTBC exhibited good linearity (coefficient of determination R^2^ = 0.9996).

The LOQ was determined to be 0.09 μg mL^−1^, which indicates that the described method involving the new sorbent can be applied for the extraction and determination of NTBC concentrations in patient blood samples.

The accuracy, expressed as the recovery of NTBC, was in the range 68.0–70.0%, while the precision of the method was in the range 3.6–9.3%.

The ME was not considered significant because its value (17%) was lower than 20%. Therefore, it was concluded that the signal of the analyte was not influenced by coextracted matrix compounds.

## 3. Materials and Methods

### 3.1. Standards and Reagents

TSP and NTBC standard (HPLC grade, ≥95%) was purchased from Sigma Aldrich (St. Louis, MO, USA).

Individual stock solutions of NTBC were prepared in methanol (MeOH) at concentrations of 1 mg/mL and stored in a refrigerator (7 °C). Working standard solutions were prepared daily by diluting the stock solutions with MeOH.

Deuterium oxide was obtained from The Radioisotope Production and Distribution Centre (Świerk, Poland).

Acetonitrile and trifluoroacetic acid (HPLC grade) were purchased from VWR (Gdańsk, Poland). Deionized water was obtained from Millipore (Burlington, MA, USA).

*N*-VPy (≥99%) and γ-MAPS (98%) were purchased from Sigma Aldrich (Saint Louis, MO, USA).

*N*,*N*-Dimethylformamide (DMF, 99.8%), dimethyl sulfoxide (DMSO, 99.7%), maleic anhydride (MAh, 99%), styrene (99%), 2,2′-azobis(2-methylpropionitrile) (AIBN, 98%), β-CD (98%), EGDMA (98%), and BIS (96%) were purchased from Chemiatrade (Gliwice, Poland). The DMF was dried over calcium hydride, filtered, and distilled under reduced pressure (8 mmHg/31 °C), and β-CD was dried in an oven at 120 °C for 24 h.

Acetone, chloroform, MeOH, and toluene (analytical grade) were purchased from ChemLand (Stargard, Poland); acetone was dried over magnesium sulphate (MgSO_4_).

Sodium dihydrogen phosphate dihydrate (NaH_2_PO_4_·2H_2_O), sodium phosphate dibasic dodecahydrate (Na_2_HPO_4_·12H_2_O), sodium bicarbonate (NaHCO_3_), sodium chloride, calcium chloride, and potassium chloride (all analytical grade) were purchased from POCH (Gliwice, Poland).

MgSO_4_ (dried, 97%) was purchased from Fisher Scientific (Hampton, NH, USA). Silica gel (0.05–0.20 mm) was purchased from Merck (Darmstadt, Germany).

### 3.2. Synthesis of β-Cyclodextrin Maleate

β-cyclodextrin maleate (β-CDMAh) was synthesized by adding maleic anhydride to β-CD (Scheme 1), according to a modified version of a published procedure [39]. Specifically, in a 50 mL round-bottom flask, 5.00 g (4.4 mmol) of dried β-CD was dissolved in 30 mL of dehydrated DMF. Then, 1.10 g (11.22 mmol) of maleic anhydride was added. The reaction was allowed to proceed in a sealed flask with continuous stirring at 80 °C for 15 h. The crude product was precipitated in acetone and subjected to continuous extraction in acetone using a Soxhlet apparatus for 48 h. The residual solvent was evaporated from the product under reduced pressure, and 4.57 g of pure product was obtained (69.2% yield). β-CDMAh was characterized by NMR spectroscopy:

^1^*H-NMR* (600 MHz, D_2_O): δ(TSP) 6.61–6.51 (m, -CH=, 7′); 5.82–5.69 (m, -CH=, 8′); 5.03 (m, 1′); 4.96 (d, *J* = 3,7 Hz, 1); 4.43 (m, 5′); 4.24 (m, one of 6′); 3.88 (m, 3′); 3.85–3.59 (m, 3,6,5, one of 6′); 3.58–3.47 (m, 2′,2,4′,4).

### 3.3. NMR Characterization of β-CD/NTBC Complexes

The interactions between the β-CD and NTBC were evaluated based on their ^1^H-NMR spectra recorded at 25 °C using a 600 MHz Varian spectrometer in D_2_O (TSP was used as an internal reference). All of the tested solutions were prepared in deuterated phosphate-buffered solution (0.1 M, pD = 8.00). The buffer pH (pD) was close to that of artificial plasma (i.e., corresponding to the pH of blood) [41]. In addition, NTBC is sparingly soluble in water (0.008 mg mL^−1^), although its solubility increases in alkaline solutions (0.5 mg mL^−1^) [42].

To make the phosphate-buffered solution with pD = 8.00, two 0.2 Mstock solutions of suitable salts were first prepared in deuterated water: solution (*A*) contained Na_2_HPO_4_·12H_2_O, and solution (*B*) contained NaH_2_PO_4_·2H_2_O. Next, 5 mL of the solution obtained by mixing solutions *A* and *B* in a 10:1 volume ratio was transferred to a 10-mL volumetric flask, which was then filled to the mark with deuterated water.

#### 3.3.1. Continuous Variation Method for Determining the Stoichiometry

Equimolar solutions (10 mM) of β-CD and NTBC were prepared in D_2_O buffer solution and distributed among 11 NMR tubes such that the molar fractions of β-CD varied between 0:1 and 1:0 (with a constant sample volume of 0.6 mL). A solution of TSP (in D2O) placed in melting-point capillary was used as an external reference. Standard ^1^H-NMR spectra were acquired with a relaxation delay of 5 s. The exact values of the molar fractions were determined based on the intensity of the appropriate signals. Job’s plots were constructed according to the chemical shifts of the aromatic proton signals of NTBC, as well as H^3^ and H^5^ of β-CD.

#### 3.3.2. 2D-ROESY

For 2D-ROESY measurements, a solution of β-CD (15 mM) and NTBC (60 mM) was prepared in deuterated phosphate buffer; no reference was added, and the chemical shifts were calibrated based on the residual water signal at 4.790 ppm [43]. ROESY experiments were carried out on a 600 MHz spectrometer with a mixing time of 500 ms.

#### 3.3.3. Titration Studies for Evaluating the Association Constants

Two stock solutions were prepared in D_2_O buffer solution: one containing only β-CD (10 mM), and the other containing β-CD (10 mM) and NTBC (40 mM). These solutions were mixed in an NMR tube such that the molar ratio of NTBC/β-CD varied from 0.0 to 4.0, while keeping the concentration of β-CD constant at 10 mM. A solution of TSP in D_2_O placed in a capillary tube was used as an external reference. Standard ^1^H-NMR spectra were acquired with a relaxation delay of 5 s. The exact values of the molar ratio of NTBC to β-CD were calculated based on the appropriate ^1^H-NMR signal integrations.

### 3.4. Modification of Silica Gel

Before the experiments, silica gel was dried at 120 °C for 24 h to remove adsorbed water on its surface.

Two methods were used to obtain activated silica gel surfaces. The first method was performed according to a report by Choi and coworkers [44]. Specifically, 10 g of silica gel was dispersed in 160 mL toluene in a 250-mL round-bottomed flask. Subsequently, 9.6 mL of γ-MAPS was added to the solution under nitrogen at 0 °C. The mixture was stirred at 80 °C for 3 days. After filtration and washing with methanol, the modified silica gel (γ-MAPS SG 1) was dried to a constant weight.

The second modification method was performed in the presence of catalytic amounts of acidic acid. Specifically, 10 g of silica gel was dispersed in 126 mL of acetone in a 250-mL round-bottomed flask. Then, 9.6 mL of γ-MAPS and four drops of acetic acid were added. The mixture was stirred at the room temperature for 4 h. After filtration and washing with acetone, the modified silica gel (γ-MAPS SG 2) was dried to a constant weight.

### 3.5. Surface Grafting of β-CDMAh on Silica Gel

Two polymers grafted on the surface of modified silica gel were tested. In all cases, β-CDMAh (synthesis described in Section 3.2) was used as the functional monomer. Polymeric sorbents were prepared using a thermally initiated free radical polymerization method. The detailed synthetic conditions are presented in Table 2.

The functional monomer was dissolved in the solvent, and then, the comonomer, crosslinking agent, and polymerization initiator were added. The mixture was transferred to a reaction vessel that contained previously weighed modified silica gel. The contents of the vessel were degassed via freeze–pump–thaw cycling in order to remove oxygen, which can inhibit the polymerization. The reaction vessel under a nitrogen atmosphere was stirred at 80 °C for 24 h. In the next step, the obtained sorbent was washed with water, methanol, and chloroform, and then dried to a constant weight.

### 3.6. Fourier Transform Infrared Spectroscopy

The obtained sorbents were characterized by FTIR. The FTIR spectra were recorded using an FTIR 6700 spectrometer (Nicolet, Thermo Fisher Scientific Inc.) equipped with a diamond crystal Smart OrbitTM accessory working in ATR mode. The FTIR spectra of the samples were measured in the range from 500 to 4000 cm^−1^ with 64 accumulations. The software OM-NIC 8.1 (Thermo Fisher Scientific Inc.) applied an advanced ATR correction of the recorded spectra.

### 3.7. SPE Procedure

First, 300 mg of silica gel and of each polymer based on modified silica gel were separately packed in 3-mL polypropylene SPE columns and secured at both ends with polyethylene frits. The sorbent was conditioned with 3 mL of methanol. Then, 1 mL of a standard solution of NTBC (20 μg/mL) in phosphate buffer (pH = 8.00) was loaded onto the column and the effluent was collected. The analyte was eluted with 1 mL of methanol, and the eluate was also collected for analysis. After the elution step, the sorbent was washed with 3 mL of methanol and dried under vacuum.

The SPE procedure was performed in three replicates for each sorbent (n = 3).

### 3.8. Chromatographic Analysis

**Figure 8:**
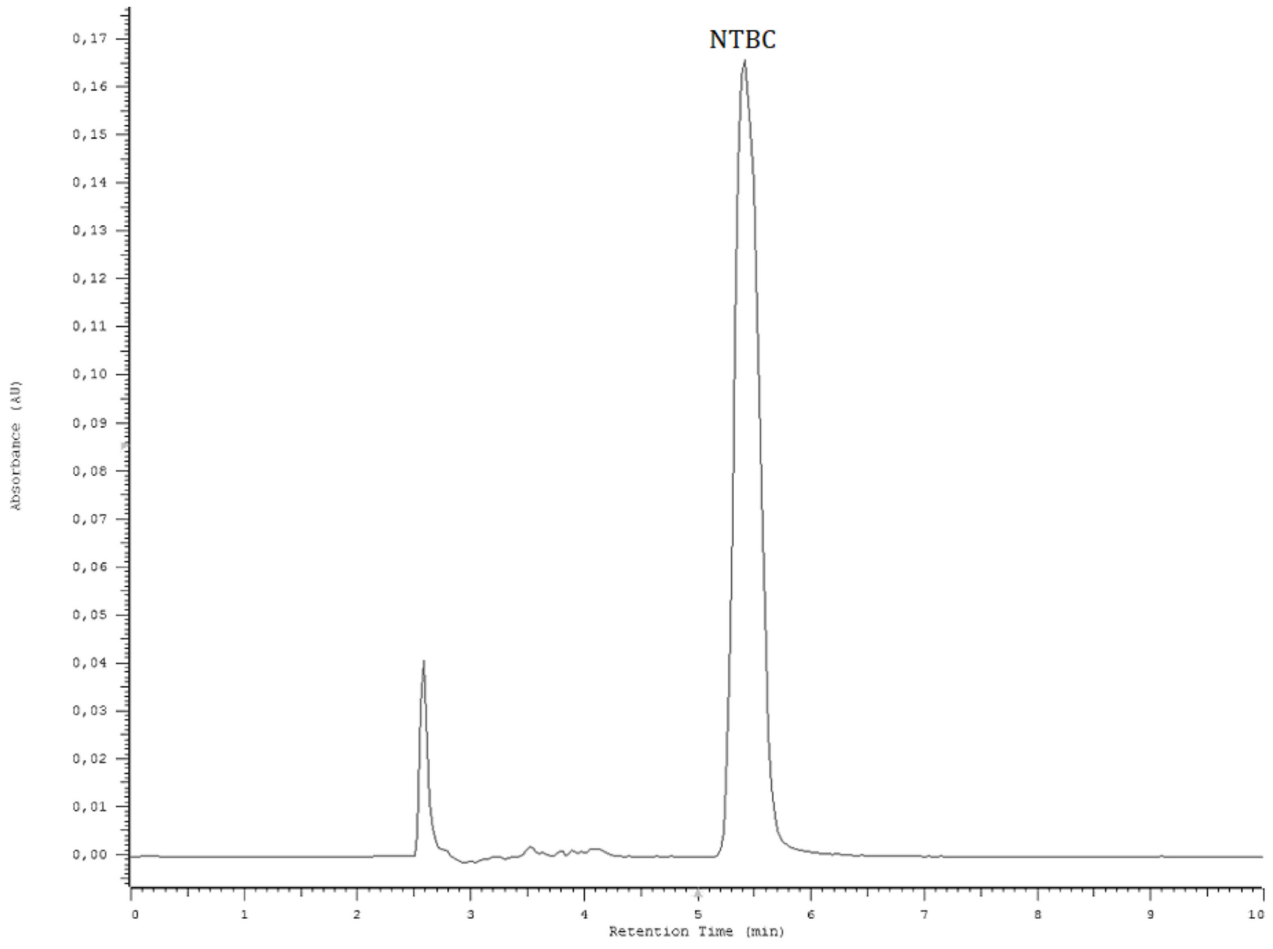
The chromatogram of NTBC standard solution prepared in methanol (c = 20 µg/mL).

The NTBC contents in the collected effluents and eluents were determined using a high performance liquid chromatograph (HPLC; Merck Hitachi, Tokyo, Japan) equipped with an L-7100 pump, a D-7000 interface, a diode array detector (DAD) L-4500A, an autosampler L-7200, and a column-thermostat Jetstream 2 Plus column. TSKgel ODS-100V (octadecyl, 150 mm × 4.6 mm, 5 µm particle size, TOSOH Bioscience, Tokyo, Japan) was used as the chromatographic column at 24 °C. The mobile phase consisted of ACN (A) and 0.05% TFA in water (B) in isocratic mode (80% A), with a flow rate of 0.5 mL/min and an analytical time of 10 min. The injection volume was 20 μL, and quantitative analysis was performed at the analytical wavelength λ_MAX_ = 271 nm. The chromatogram of NTBC (c = 20 μg/mL) was presented in Figure 8.

The chromatographic analyses of effluents and eluates were performed in three replicates (n = 3).

### 3.9. Analytical Method Validation

The analytical method was validated based on the sorbent that achieved the highest analyte RE (the highest NTBC content in the eluate). The matrix comprised artificial plasma prepared according to a method reported by Liu and coworkers [41], without bovine serum albumin (BSA).

Six calibration points in the range of 1–25 μg/mL were prepared in artificial plasma (in three repetitions; n = 3) to obtain information regarding the linearity, sensitivity (LOQ), and ME of the method. Moreover, the NTBC recovery, and the intraday and interday precision (RSD) at 5 and 15 μg/mL in the artificial plasma were evaluated (n = 3).

## 4. Conclusions

A new polymeric sorbent containing derivatives of β-cyclodextrin grafted on silica gel was developed, and it was successfully employed for the extraction of NTBC from model physiological fluids. Its demonstrated effectiveness for NTBC extraction from artificial plasma was a result of the formation and geometry of an inclusion complex comprising NTBC and β-CD.

The elaborated SPE-HPLC-DAD method involving the novel polymeric sorbent containing β-cyclodextrin derivatives grafted on silica gel exhibited good linearity, sensitivity, accuracy, and precision and is thus deemed suitable for the determination of NTBC in patients’ blood samples.

During treatment with NTBC, the concentration of L-tyrosine may increase in patients’ blood due to inhibition of the metabolic pathway of this amino acid by drug [3].

L-tyrosine, similarly to NTBC, has a tendency to form inclusion complexes with β-CD. The values of association constants reported in the literature, K = 105.00 M^−1^ [45] and K = 60.25 M^−1^ [46], are close to the values for the complex of β-CD with NTBC, determined in the present work. Therefore, the selectivity of the developed sorbents will be studied in the future, especially in respect to the L-tyrosine.

## Figures and Tables

**Table 1:** Validation parameters for the elaborated method involving the new sorbent SG2_St_β-CDMAh_EGDMA.

Analyte	Range (μg mL^−1^)	Linearity (R^2^)	LOD (μg mL^−1^)	LOQ (μg mL^−1^)	ME (%)	Recovery ± RSD (%)		Interday Precision (%RSD)	
						5 μg mL^−1^	15 μg mL^−1^	5 μg mL^−1^	15 μg mL^−1^
NTBC	1.0–25.0	0.9996	0.03	0.09	17.0	68.0 ± 5.8	70.0 ± 3.8	9.3	3.6

**Table 2:** Detailed conditions for the sorbent syntheses.

Sorbent	Silica Gel	Mass (g)	Functional Monomer	Mass (g)	Solvent	Volume (mL)	Comonomer	Number of Moles (g/mmol)	Cross-Linking Agent	Content (g/mmol)	Polymerization Initiator	Mass (g)
SG1_*N*-VPy_β-CDMAh_BIS	SG 1	3.0	β-CDMAh	1.0	H_2_O	7.6	*N*-VPy	0.6/5.0	BIS	0.05/0.3	AIBN	0.023
SG2_*N*-VPy_β-CDMAh_BIS	SG 2				H_2_O		*N*-VPy	0.6/5.0	BIS			
SG2_St_β-CDMAh_EGDMA					DMSO		styrene	0.29/2.7	EGDMA	0.19/1.0		

## Data Availability

Not available.

## Samples Availability

Samples of the compounds are not available from the authors.
